# Bullous pemphigoid and neurodegenerative diseases: a study in a setting of a Central European university dermatology department

**DOI:** 10.1007/s40520-015-0459-4

**Published:** 2015-09-29

**Authors:** Paweł Pietkiewicz, Justyna Gornowicz-Porowska, Monika Bowszyc-Dmochowska, Paweł Bartkiewicz, Marian Dmochowski

**Affiliations:** Autoimmune Blistering Dermatoses Section, Department of Dermatology, Poznan University of Medical Sciences, 49 Przybyszewski Street, 60-355 Poznan, Poland; Cutaneous Histopathology and Immunopathology Section, Department of Dermatology, Poznan University of Medical Sciences, Poznan, Poland

**Keywords:** Bullous pemphigoid, Neurodegenerative diseases, Aging

## Abstract

Bullous pemphigoid (BP) is an autoimmune blistering dermatosis of the elderly mediated by IgG and IgE antibodies to skin hemidesmosomal proteins, BP180 and/or BP230, that occur physiologically also in neuronal tissue. It was reported that BP is associated with neurodegenerative diseases (ND). We performed a retrospective study in a setting of a Central European university dermatology department on prevalence of ND in 94 BP patients. 26 out of 94 BP patients had at least one ND. ND included: Parkinson’s disease, dementia, stroke, hear loss, tinnitus, blindness, vertigo, neurosyphilis, systemic sclerosis, and epilepsy. Since population aging is conceivably responsible for the rising number of BP cases as a result of immunosenescence-related phenomena, the plausible BP-specific immunopathogenetic relationship between BP and ND deserves to be further experimentally explored.

## Introduction

Bullous pemphigoid (BP) is an autoimmune blistering dermatosis common among elderly octogenarians and older. IgG- and IgE-mediated autoimmunity to hemidesmosomal proteins, BP180 and/or BP230, and mixed neutrophil/eosinophil infiltrate clinically results in forming tense-top subepidermal blisters on urticariform plaques, erythematous or noninflamed skin, predominantly on the flexural aspects of limps and on the trunk [1–3]. The neuronal forms of these proteins, BP180 (type XVII collagen, BPAG2) and BP230 (dystonin, BPAG1n, BPAG1a), occur physiologically in neuronal tissue and are fundamental for retainment of neuro-cytoskeleton organization. Transmembrane protein BP180 seems to be associated with synapse stabilization in central nervous system and synaptic plasticity of the brain, whereas intracellular protein BP230 anchors the neural intermediate filaments to the actin cytoskeleton, microtubule and microfilament cytoskeletal networks with each other and to distinct cell membrane sites, acting also as a scaffold for signaling proteins that regulate the cytoskeleton alteration [4].

Dystonin (DST) gives many transcript variants of BPAG1 plakin family proteins (due to alternative splicing phenomenon), whereas their role is mostly unclear. There are three main isoforms of BPAG1: BPAG1e (BP230) in stratified epithelia, BPAG1a (or BPAG1n) in neuronal tissue and BPAG1b in striated muscle tissue. Inactivation of DST results in neurodegeneration and blistering in mice and hereditary autonomic sensory neuropathy in human [5–7]. It was reported that BP is associated with, and in that cases is usually preceded by, neurodegenerative diseases (ND) (e.g. Parkinson’s disease, dementia, stroke, epilepsy, multiple sclerosis or even neurosyphilis) [8–11]. It was estimated in a British study that in about 72 % BP patients ND preceded the disease by a mean of 5.5 years [12]. Interestingly, it was recently shown that dementia and Parkinson’s disease patients without BP may also develop cerebrospinal fluid (CSF) anti-BP180 antibodies to non-pathogenic intracellular and extracellular domains and, rarely, to pathogenic NC16A domain of BP180 [13]. Moreover, it was proved that BP patients with ND disease have anti-BP230 serum autoantibodies that bind both epithelial (BPAG1e) and neuronal (BPAG1a) isoforms [14]. Cases of psoriasis preceding autoimmune blistering dermatoses, particularly BP, were reported in the literature, including a report from our university department [15]. We have recently seen a male octogenarian with psoriasis lasting many decades that underwent erythrodermic exacerbation with extensive scaling 2 weeks before presentation followed by development of numerous intensely itchy blisters especially on palms and soles just a week before presentation. On the ground of clinical picture and results of laboratory tests, dyshidrotic BP coexistent with erythrodermic psoriasis was diagnosed. Therefore, conceivably, progressing conformational modifications, in numerous combinations, of neuronal, epidermal or both neuronal and epidermal isoforms of BP antigens triggered by a plethora of stimuli may induce the development of BP-type autoimmunity and finally clinically overt BP.

The objective of this study was to analyze the prevalence of ND in BP patients in a setting of Central European dermatology department.

## Materials and methods

The medical history of 94 BP patients diagnosed at molecular level with direct immunofluorescence, indirect immunofluorescence mosaic (IIFm) (Euroimmun, Germany) and ELISA (Euroimmun, Germany) [26 BP patients with ND (BP+ND) and 68 BP patients without ND (BP–ND)] hospitalized in Central European university dermatology department between December 2006 and May 2015 (BP) were reviewed for the presence of ND records. ND included: Parkinson’s disease, dementia, stroke, hear loss, tinnitus, blindness, vertigo, neurosyphilis, systemic sclerosis, and epilepsy. The study group is described in the Tables 1 and 2.

**Table 1:** Studied groups

	BP (*n* = 94)
M/F ratio	0.59
Mean age ± SD	75 ± 11
Patients with more than one ND	3

*BP* bullous pemphigoid; *n* number of cases; *M/F ratio* male to female ratio; *ND* neurodegenerative disease; *SD* standard deviation

**Table 2:** Neurodegenerative diseases in studied groups

ND	BP (*n* = 94)
Stroke	12
Dementia	5
Parkinson’s disease	5
Hear loss	1
Vertigo	2
Blindness	1
Tinnitus	1
Systemic sclerosis	1
Epilepsy	1
Neurosyphilis	1

*BP* bullous pemphigoid; *n* number of cases; *ND* neurodegenerative disease

## Results

The prevalence of ND in the analyzed group was 27.66 %, stroke—12.77 %, Parkinson’s disease—5.32 %, dementia—5.32 % and number of patients with more than one ND reached 3.19 %.

## Discussion

BP is the most frequent autoimmune blistering dermatosis (incidence 7–43 per million population per year), debilitating condition affecting mostly the elderly [16, 17]. Recently re-estimated risk of death in BP patients seems to be more than two- to sixfold higher than in general population [18, 19], while the 1-year survival is reported to be 62 % [19]. Because of population ageing, BP and associated disorders become burning socioeconomic problem [18, 19]. Thus, there is an urgent need for in-depth studies concerning disease mechanisms. The course of BP in patients with anti-BP180 IgG, and thus ND rate, may differ from that seen in individuals with solely anti-BP230 IgG (pemphigoid anti-BP230) or with coexisting anti-BP230 and anti-BP180 IgG [20]. In our issue-probing retrospective immunopathologic study, the occurrence of ND in BP has not been related to levels of serum IgG antibodies to either BP180 or BP230 [21]; nevertheless, detailed assessment of cerebrospinal fluid antibodies to BP180 [22] and BP230 could be more productive in that respect. The knowledge about the pathogenetic mechanisms interlinking ND and BP, as well as immunologic features in these patients remains scant; yet several hypotheses were coiled to elucidate this phenomenon. It was speculated that ND patients received drugs (myorelaxants, neuroleptics, aldosterone antagonists) that are known triggers of BP, yet no correlation was reported between the intake time and clinical onset of BP [8]. Other speculations concerned the possibility of dermal–epidermal junction (DEJ) destruction with subsequent antigen exposition and immunomodulation due to the development of decubitus ulcers in bed-ridden ND patients, role of stress and finally age-related immunologic dysfunction resulting in autoimmunization as both neuronal tissue and skin derive from neural crest [21]. Thus, immunosenescence may be the link between examined disorders. Remodeling of innate immunity and clonotypical immunity (significant changes in the function of T cells) as well as occurrence of chronic inflammatory process may promote tissue degeneration in BP and ND [23]. The genetic alteration of BPAG1a on mouse model resulted in the accumulation of intermediate filaments in motor neurons prior to neurological degeneration and dystonia. This intermediate filaments’ accumulation was hypothesized to produce loss of tolerance to BPAG1a and giving cross-reactiveness with the epidermal BPAG1e [24].

The incidence of ND in our BP patients (27.66 %) seems to be in the middle part of dispersion reported in recent studies from United Kingdom, France, Czech Republic and Brazil: 22–46 % [9, 12, 17, 24, 25]. The differences between studies may derive from genetic and geographical factors. There is also a possibility that some BP-ND patients remain underdiagnosed, as the awareness about the linkage might be still low among dermatologists, who may marginalize non-dermatological conditions or simply neglect them in medical records. The incidence of cerebrovascular incident in BP patients (12.77 %) was similar to the one noted by the French group (15 %), but lower than in the United Kingdom (44.4 %) [12, 17, 24]. It is possible, that apart from genetic susceptibility, dietary habits and different living conditions (including post-war period) and high death rate observed in stroke patients in Poland might play a role in the number of reported cerebrovascular incidents. Parkinson’s disease was present in 5.32 % of our BP patients in comparison to 9 % in France and 30 % in United Kingdom [12, 17, 24]. Dementia rate in the studied BP group reached 5.32 %. A report on populational study in Midwestern Poland estimated that in people >65 years dementia was observed in 5.7 % [26] in what seems to be in the lower part of dispersion of the epidemiologic data in this geographic region (in contrast to 13 % in United Kingdom) [12] and corresponds with our findings. The low rate is speculated to be caused by underdiagnosing of dementia [27]. The study by Langan et al. reported threefold risk of developing BP in patients with Parkinson’s disease and dementia, whereas twofold risk in patients with stroke and epilepsy, comparing to healthy population [9].

**Fig. 1:**
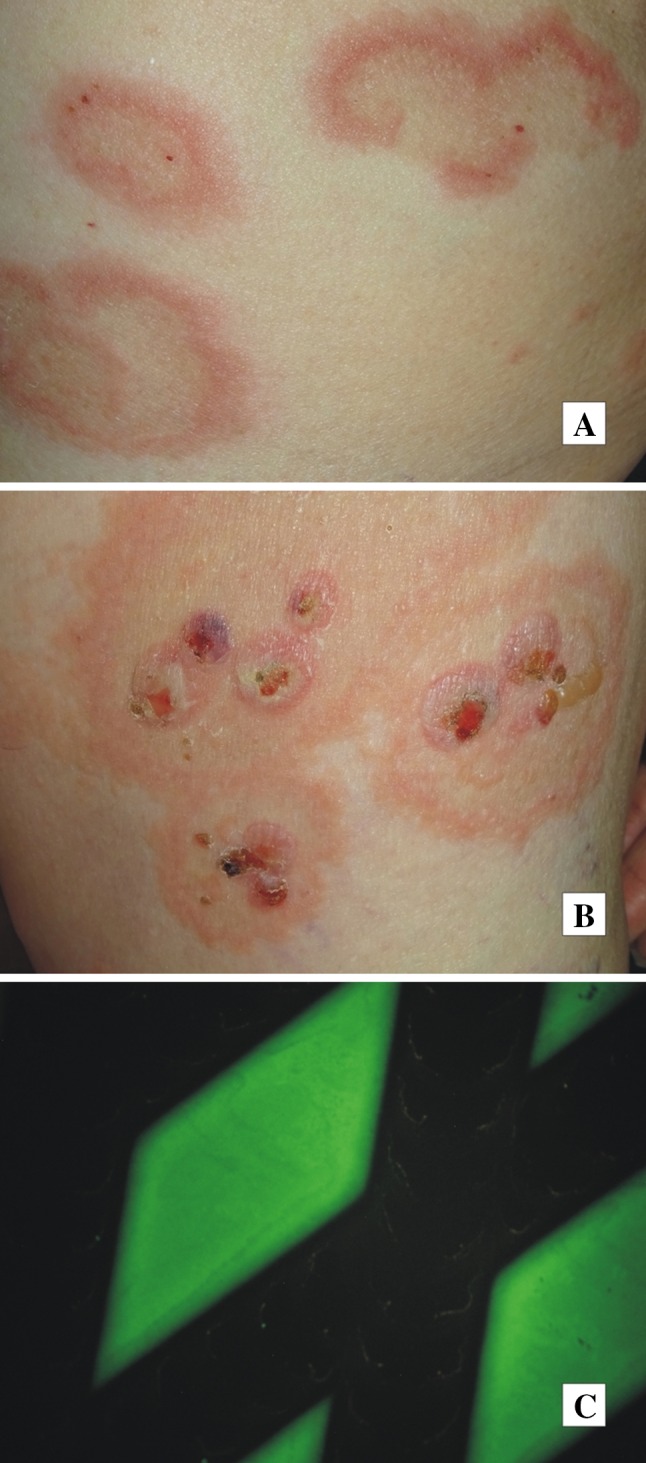
Bullous pemphigoid coexistent with late syphilis. Erythema annulare centrifugum (deep variety)-like eruption on the trunk (**a**). Ruptured blisters on erythematous base affecting medial surface of a thigh (**b**). Indirect immunofluorescence mosaic: serum IgG autoantibodies against the tetrameric BP180 NC16A spots (*antigen dots*) (**c**)

The pathogenetic ND-related mechanisms in BP have not been elucidated yet; yet they possibly involve autoantigens common in central nervous system and epidermis. We have recently seen a middle-aged female, much younger than stereotypical octogenarian with BP, with coexistent BP clinically resembling a deep variety of erythema annulare centrifugum (Fig. 1a, b) and marginally symptomatic late syphilis causing memorization impairment [28]. Importantly, both BP and syphilis were diagnosed at the usable molecular level with ELISA and IIFm for serum IgG antibodies to BP180 (Fig. 1c), and chemiluminescent microparticle immunoassay for serum antibodies to *Treponema pallidum* recombinant antigens (TpN15, TpN17, TpN47). Such coexistences may be yet another argument for experimentally exploring plausible BP-specific immunopathogenetic links between BP and ND.

## Conclusion

Although BP is relatively rare disease, patients should be routinely screened for underlying/concomitant ND. On the other hand, patients with ND should be carefully followed-up for BP development. Since population aging is conceivably responsible for rising number of BP cases, the plausible BP-specific immunopathogenetic relationship between BP and ND deserves to be further experimentally explored.
